# Functional left atrial CMR parameters are early predictors of left atrial alterations in hypertension and strongly associated with lv remodeling

**DOI:** 10.1186/1532-429X-17-S1-P355

**Published:** 2015-02-03

**Authors:** Zainab Raissuni, Morgane Evin, Elie Mousseaux, Phillipe Cluzel, Nadjia Kachenoura, Alban Redheuil

**Affiliations:** 1 Faculty of Medicine of Tangier, Tangier, Morocco; 2 Laboratoire d'Imagerie Fonctionnelle, Paris, France; 3 Radiology departement HEGP, Paris, France

## Background

Left atrial (LA) structural and functional changes are determinant steps on the pathway toward heart failure with preserved ejection fraction in hypertensive (HT) patients. Most LA studies are based on LA volumes evaluation in CMR and echocardiography or on echocardiographic speckle tracking estimation. Since LA functional evaluation by CMR is now emerging we aimed at 1) evaluating LA function by CMR in controls and hypertensive patients (moderate and severe HT) using feature tracking technique on conventional cine data; 2) evaluating the relationship between the components of LA function (reservoir, conduit, atrial contraction) and left ventricular (LV) remodeling.

## Methods

We studied 22 patients with HT (12 moderate, 9 resistant) matched with 20 healthy individuals for age and gender. Steady state free precession (SSFP) CMR cine loops were acquired in three long axis views and a custom automated feature tracking tool was used to estimate: global longitudinal strain (Sl) and strain rate (SRl), radial motion fraction (MrR) and relative velocities (Vr) as well as LA volumes using a multi plane area-length method. LA functional parameters were defined for reservoir (R), conduit (E) and LA contraction (A) phases. The LV mass index, LV wall thickness and LV ejection fraction were measured from short-axis images using the Qmass software.

## Results

Maximal indexed LA volumes were in the normal range in HT patients and comparable to controls (42.4±18.3mL/m2 *vs.* 44.5±13.1mL/m2). Alteration of LA function in hypertension was highlighted by a significant reduction in longitudinal strain and motion fraction (p<0.03) between moderate and severe HT patients (Sl_R_: 19.0±3.8% *vs.* 15.2±4.5%, p=0.04, Mr_R_ : 23.1±5.2 *vs.* 17.6±5.7, p=0.03). LA reservoir and LA contraction longitudinal strains were lower in resistant HT patients as compared to moderate HT (p<0.04) and controls (p<0.07, ns). Moreover, longitudinal strain rates and relative velocities were significantly reduced for reservoir and contraction phases in severe HT *vs.* controls (SRl_A'_:-1.0±0.3 *vs.* -1.6±0.6, p=0.007 and Vr_A'_: -1.1±0.4 *vs.* -1.7±0.7, p=0.02) and severe *vs.* moderate HT (Vr_A'_: -1.1±0.4 vs -1.8±0.7, p=0.02). However, there was no significant difference in LA conduit phase strains and strain-rates between groups.

Finally, reduced reservoir longitudinal strains were associated with LV hypertrophic remodeling as indicated by significant associations with thickness (r=-0.41, p=0.007), indexed LV mass (r=-0.32, p=0.04) and LV mass/end-diastolic volume (r=-0.53, p<0.001).

## Conclusions

CMR-measured functional LA parameters were altered in severe hypertensions even with normal LA volumes. This suggests that functional LA alterations may precede significant LA dilation. Moreover, LA functional alterations were strongly associated with LV remodeling and might be accordingly useful for therapeutic management in the setting of hypertension.

## Funding

N/A.

